# Albumin and multiple sclerosis: a prospective study from UK Biobank

**DOI:** 10.3389/fimmu.2024.1415160

**Published:** 2024-06-10

**Authors:** Ke Chen, Chunyu Li, Bi Zhao, Huifang Shang

**Affiliations:** ^1^Department of Neurology, Laboratory of Neurodegenerative Disorders, National Clinical Research Center for Geriatrics, West China Hospital, Sichuan University, Chengdu, Sichuan, China; ^2^Department of Neurology, West China School of Public Health and West China Fourth Hospital, Sichuan University, Chengdu, China

**Keywords:** albumin, multiple sclerosis, microalbuminuria, protective effect, UK Biobank

## Abstract

**Background:**

Multiple sclerosis (MS) is a chronic inflammatory disease affecting the central nervous system. While previous studies have indicated that albumin, the primary protein in human plasma, may exert influence on the inflammatory process and confer beneficial effects in neurodegenerative disorders, its role in the context of MS has been underexplored. Here, we aimed to explore the link between albumin and the risk of MS.

**Methods:**

Employing data from the UK Biobank, we investigated the association between baseline levels of serum and urine albumin and the risk of MS using Cox proportional hazards regression analysis.

**Results:**

A higher baseline level of serum albumin was associated with a lower risk of incident MS (HR=0.94, 95% CI: 0.91–0.98, P=7.66E-04). Subgroup analysis revealed a more pronounced effect in females, as well as participants with younger ages, less smoking and deficient levels of vitamin D. Conversely, no association was identified between baseline microalbuminuria level and risk of incident MS.

**Conclusion:**

Higher serum albumin level at baseline is linked to a reduced risk of MS. These results contribute to an enhanced understanding of albumin’s role in MS, propose the potential use of albumin as a biomarker for MS, and have implications for the design of therapeutic interventions targeting albumin in clinical trials.

## Introduction

Multiple sclerosis (MS) is a chronic, immune-mediated inflammatory demyelinating disease of the central nervous system (CNS), in which the immune system attacks the protective sheath (myelin) that covers nerve fibers and causes communication problems between your brain and the rest of your body (1). Currently, there’s no effective cure for MS, though several treatments could help control the condition and alleviate symptoms (2). Multiple risk factors contribute to the risk of MS, such as smoking, low vitamin D level, and obesity (3). However, these factors only account for a modest proportion of the variability in disease risk. The identification of biomarkers capable of predicting disability progression, monitoring ongoing disease activity, and assessing treatment response is crucial for making informed decisions regarding the management of MS (4).

Molecular biomarkers represent a pivotal advancement in evaluating therapy response and potential side effects in MS (5). Among these biomarkers, albumin, the main protein in the blood plasma produced in the liver, is closely implicated in the pathogenesis of MS. Given its high concentration in the plasma, albumin would be expected to access CNS tissue following the breakdown of the blood-brain barrier (BBB) that occurs during MS. An elevated level of albumin in the cerebrospinal fluid (CSF), or an elevated albumin quotient, is thought to be a measure of blood-CSF dysfunction in MS (6), and serve as an indicator of BBB permeability. Increased albumin quotient at clinical onset was suggested to be associated with increased brain atrophy and greater disability in patients after first clinical event suggestive of MS (7). Furthermore, serum albumin exhibits antioxidant properties and may exert a protective effect on the disease course by acting as a target for reactive molecules that would otherwise have greater access to damaging more crucial biomolecules (8, 9). Collectively, these cumulative evidence underscores the essential role of albumin in the pathogenesis of MS. Contradictory results, however, have been documented. For instance, previous retrospective study among 40 patients with MS attack and 40 controls found no difference in terms of albumin levels (10). Nevertheless, it is important to recognize that observational studies may be susceptible to limitations such as small sample sizes and potential confounding factors (11). Moreover, these studies predominantly encompass prevalent cases and offer limited insights into whether prediagnostic albumin levels hold predictive value for the future risk of MS. Therefore, further research is imperative to explore the association between albumin and MS.

In this context, we analyzed the association between serum and urine albumin level and the risk of MS based on longitudinal data from the United Kingdom (UK) Biobank. Our results revealed that a higher baseline serum albumin level was significantly associated with a reduced risk of incident MS.

## Methods

### Participants

The study utilized resources from the UK Biobank, a prospective population-based cohort study that enrolled approximately 500,000 residents aged between 39 and 72 years, recruited between 2006 and 2010 in the UK (12). The data collection process was comprehensive and included the administration of questionnaires, physical measurements, blood sample assays, genotyping, and the integration of electronic health data gathered from participants across 22 assessment centers throughout the UK. All participants provided informed consent for data collection and linkage. The UK Biobank received ethical approval from the NHS National Research Ethics Service (16/NW/0274). This research was conducted using the UK Biobank resource under application number 98992 (13).

### Exposures

The exposure of interest was serum albumin level at baseline. Blood samples were collected from participants at the final station of the baseline visit, and serum was prepared and stored at a temperature of -80°C until assayed. Serum albumin levels were measured by enzymatic analysis on a Beckman Coulter AU5800 machine using a kinetic modification of the Jaffe procedure. Details regarding the assay and quality control procedures could be found in the UK Biobank website (biobank.ndph.ox.ac.uk/ukb/ukb/docs/serum_biochemistry.pdf). In addition, we analyzed baseline urine microalbumin level as an exposure. Urine samples were collected at baseline, and urine albumin measurement was carried out on a single Beckman Coulter AU5400 clinical chemistry analyzer using the manufacturer’s reagents and calibrators.

### Outcomes

The primary outcome we focused on was new diagnosis of MS. Data were collected from multiple sources including participants’ self-reported diagnoses obtained through verbal interviews and electronic health records, which encompassed hospital admissions data, primary care data, and death registers. The coding systems employed were the International Classification of Diseases, Tenth Revision (ICD-10) codes, and the Read coding system codes (**Supplementary Table 1**). The follow-up for MS incidence was conducted until November 09, 2022. The baseline period was defined as the date of recruitment, while the end of the follow-up period was defined as the date of MS incidence, date of death, or end of follow-up, whichever occurred first. To address concerns regarding reverse causality, we excluded participants with prevalent MS at the time of study enrollment. Additionally, participants with a short latency period of two years or less between the initial sampling and the MS diagnosis were also excluded, with the aim of minimizing the possibility of reverse causality (14).

### Statistical analysis

We employed Cox proportional hazards models to derive hazard ratios (HRs) and 95% confidence intervals (CIs) to assess the associations between baseline albumin level and risk of MS. Interaction analyses were performed by incorporating interaction terms into the Cox models. Our analyses were conducted initially for the entire study population, and then stratified by different demographic factors such as gender (males and females), age (<65 years and ≥65 years), BMI (<25 kg/m^2^ and ≥25 kg/m^2^) and smoking (Yes, Occasionally, No). We utilized two distinct models for our analyses. In Model 1, the minimally adjusted model, we adjusted for the fundamental demographic variables of sex and age since the incidence of MS can vary based on these factors. In Model 2, the fully adjusted model, we included additional covariates to account for socioeconomic status (such as the Townsend deprivation index and education) and lifestyle factors (including BMI, smoking status, and alcohol consumption, physical activity, vitamin D) that have been previously linked to the risk of MS (**Supplementary Methods**). To ensure the robustness and accuracy of our results, we excluded participants with missing values for any of the variables included in the models. Continuous variables were presented as median (interquartile range), and categorical variables were presented as percentages (numbers). Wilcoxon test was used to compare the continuous variables, while Fisher’s exact test to compare categorical variables. Statistical analyses were performed in R v3.5.3.

## Results

### Population characteristics

**Figure 1:**
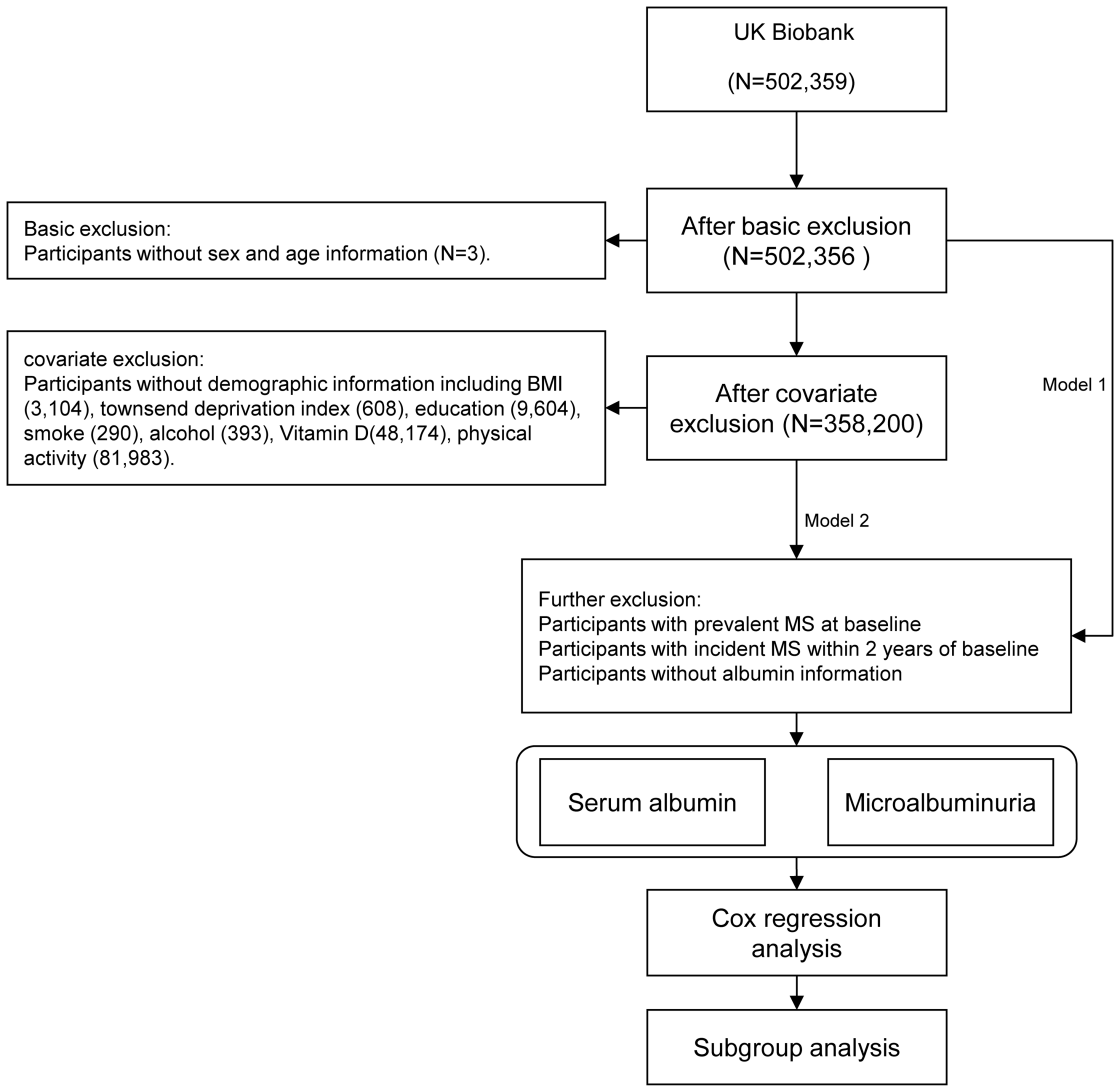
Schematic overview of the study design.

The UK Biobank recruited a total of 502,359 participants. After excluding participants without age (N=3) and sex (N=0) information, a total of 502,356 individuals remained for regression analysis in Model 1. After further filtering by demographic data, a total of 358,200 individuals remained for regression analysis in Model 2, including 173,376 (48.4%) males (**Figure 1**).

### Associations of albumin with MS

**Figure 2:**
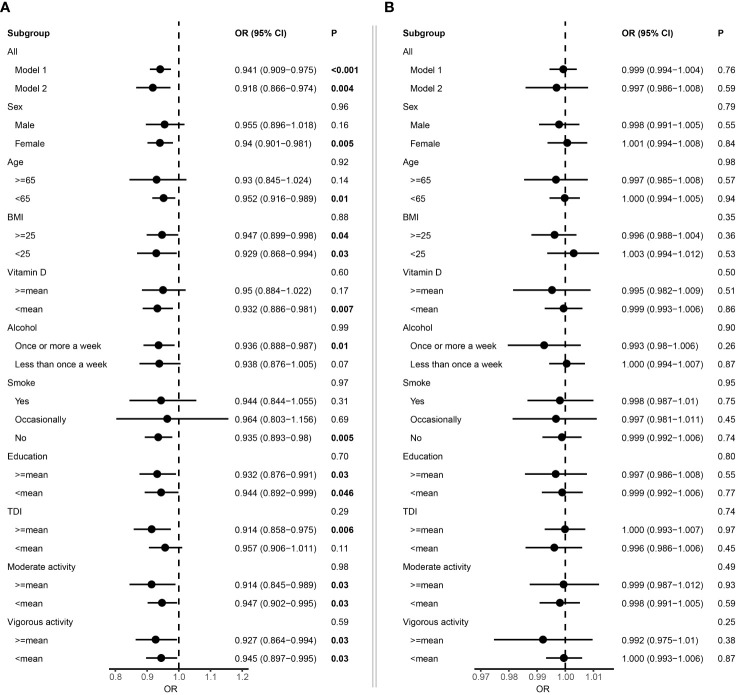
Forest plot showing the association between albumin and multiple sclerosis. **(A)** Results from Cox proportional hazards regression and subgroup analyses of the association between serum albumin and multiple sclerosis. Error bars indicate 95% confidence intervals. Model 1 adjusted for age and sex. Model 2 adjusted for additional covariates including Townsend deprivation index, education, BMI, smoking status, alcohol consumption, physical activity, and vitamin D. P values for each covariate were derived from interaction analysis, while P values for each subgroup were derived from the Cox proportional hazards regression analysis. **(B)** Results from Cox proportional hazards regression and subgroup analyses of the association between microalbuminuria and multiple sclerosis. Bold P value denotes P value < 0.05.

We firstly employed Cox proportional hazard regression analysis to investigate the longitudinal associations between baseline albumin level and incident MS. During a mean follow-up of 13.3 years, 539 participants developed MS, after excluding patients with MS at baseline (N=2,011) or diagnosed within 2 years of initial sampling (N=48). In general, individuals who developed MS were younger, had lower vitamin D level and smoked more (**Table 1**). After adjustment for fundamental covariates in Model 1, per SD increment of serum albumin was associated with a lower risk of incident MS (HR=0.94, 95% CI: 0.91–0.98, P=7.66E-04) (**Figure 2A**). These results remained consistent and stable after further adjustment for additional covariates in Model 2, with the same trend observed (HR=0.92, 95% CI: 0.87–0.97, P=4.41E-03). Additionally, we did not detect interaction among albumin and each covariate with respect to the risk of incident MS. In the subgroup analyses, the protective effect was more pronounced in females, as well as individuals with less smoking, lower vitamin D level and younger ages (**Figure 2A**). In contrast, urine microalbumin was not associated with risk of incident MS (**Figure 2B**).

**Table 1:** Baseline characteristics of the UK Biobank cohort by disease status.

Baseline Characteristic	All			Female			Male		
	cases	non-cases	P	cases	non-cases	P	cases	non-cases	P
Sociodemographic									
Age (years)	54 (14)	58 (13)	**<0.001**	53 (14)	57 (13)	**<0.001**	55 (14)	58 (14)	**0.025**
Education (years)	13 (10)	13 (10)	0.433	13 (10)	13 (10)	**0.018**	13 (10)	15 (10)	0.132
BMI (kg/m^2^)	26.74 (6.04)	26.62 (5.66)	0.524	26.15 (6.66)	25.89 (6.11)	0.980	27.63 (4.52)	27.23 (5.02)	0.968
Townsend deprivation index	-2.12 (4.05)	-2.22 (4.03)	0.310	-2.05 (4.31)	-2.20 (4.00)	0.160	-2.26 (3.55)	-2.26 (4.06)	0.842
Vitamin D (nmol/L)	39.45 (29.33)	47.30 (30.00)	**<0.001**	37.90 (27.05)	47.40 (30.00)	**<0.001**	42.20 (31.95)	47.10 (29.90)	**0.016**
Moderate activity (minutes/week)	360 (1160)	480 (1080)	**0.001**	360 (1120)	480 (1080)	0.055	240 (1200)	480 (1080)	**0.005**
Vigorous activity (minutes/week)	80 (720)	240 (960)	**0.006**	80 (720)	160 (720)	0.056	80 (960)	240 (960)	0.154
Smoke: No	0.80 (335)	0.90 (320709)	**<0.001**	0.85 (226)	0.91 (168060)	**<0.001**	0.72 (109)	0.88 (152649)	**<0.001**
Occasionally	0.05 (22)	0.03 (9864)	**0.004**	0.03 (9)	0.02 (3891)	0.194	0.09 (13)	0.03 (5973)	**0.002**
Mostly	0.15 (61)	0.07 (25990)	**<0.001**	0.12 (32)	0.06 (11732)	**<0.001**	0.19 (29)	0.08 (14258)	**<0.001**
Alcohol: Never	0.10 (42)	0.07 (25900)	**0.037**	0.10 (28)	0.09 (15894)	0.276	0.09 (14)	0.06 (10006)	0.078
Special occasions	0.12 (51)	0.11 (37872)	0.301	0.15 (41)	0.14 (25912)	0.538	0.07 (10)	0.07 (11960)	1.000
1-3 times a month	0.15 (63)	0.11 (39038)	**0.010**	0.16 (42)	0.13 (23812)	0.172	0.14 (21)	0.09 (15226)	**0.042**
1-2 times a week	0.26 (110)	0.26 (92304)	0.823	0.27 (73)	0.26 (47795)	0.625	0.25 (37)	0.26 (44509)	0.780
3-4 times a week	0.18 (77)	0.24 (85732)	**0.007**	0.19 (51)	0.21 (39421)	0.371	0.17 (26)	0.27 (46311)	**0.007**
Daily	0.18 (75)	0.21 (75717)	0.106	0.12 (32)	0.17 (30849)	**0.040**	0.28 (43)	0.26 (44868)	0.459
Biomarker									
Serum albumin (g/L)	44.81 (3.52)	45.20 (3.43)	**0.002**	44.47 (3.41)	44.92 (3.41)	**0.006**	45.54 (3.32)	45.51 (3.41)	0.645
Urine microalbumin (mg/L)	11.90 (8.93)	11.40 (10.70)	0.703	12.10 (9.45)	11.10 (9.20)	0.106	11.50 (8.30)	11.80 (12.50)	0.315

Continuous variables were presented as median (IQR); Categorical variables were presented as percentages (numbers). Wilcoxon test was used to compare the continuous variables, and Fisher’s exact test to compare categorical variables. IQR, interquartile range. Bold P value denotes P value < 0.05.

## Discussion

Prior epidemiological investigations have presented evidence pointing to a potential link between albumin level and risk of MS, though the findings have displayed inconsistencies (7, 10, 15). In the current study, we examined the association between baseline albumin levels in serum and urine and the risk of MS. Our results demonstrated that higher serum albumin level was associated with a decreased risk of MS. These findings suggested the implication of targeting serum albumin as a potential treatment option for MS in future clinical trials.

In the current cohort, individuals who developed MS tended to be younger, had lower vitamin D levels, and were more likely to smoke. The average age of the population was 56.54 years, older than the typical average age at onset of patients with MS (16, 17). Consistently, previous epidemiological studies have suggested association between vitamin D insufficiency and increased risk of MS (18, 19), as well as smoking and higher risk of MS (20). Serum albumin is the primary plasma component affecting oncotic pressure, transporting fatty acids, carrying hormones, influencing drug pharmacokinetics, binding metals and heme, and acting as an anti-oxidant. Recent investigations revealing amyloid precursor protein expression in axons around plaques in MS, along with the correlation of amyloid-β (Aβ) with various MS stages, unequivocally highlight the pivotal role of amyloid in MS pathogenesis (21). Several endogenous proteins interacting with Aβ can modulate its amyloidogenic process, including serum albumin, a key endogenous inhibitor of Aβ aggregation (22). Serum albumin binds to Aβ and facilitates Aβ efflux from the cerebrospinal fluid to plasma. In addition, studies have established the involvement of reactive oxygen species (ROS) and reactive nitrogen species (RNS) in MS pathogenesis, with elevated markers of ROS and RNS observed in MS (23). Considering the high concentration of albumin in plasma and the compromised blood-brain barrier during disease, albumin is anticipated to be an abundant substrate for ROS and RNS in MS, suggesting a potential protective effect (9). Furthermore, albumin acts as an antioxidant, mitigating excessive oxidant stress induced by inflammation in aging neuronal cells (24). Both oxidative stress and inflammation have been proposed to play a significant role in the MS pathogenesis. Given the ability of antioxidants to reduce inflammatory responses, it is plausible to hypothesize that albumin may confer beneficial effects against MS. In the subgroup analysis, we observed a more pronounced protective effect of serum albumin in females, as well as individuals with lower levels of smoking, vitamin D, and younger ages. Serum albumin concentration tends to decrease with age, particularly in females at a faster rate than in males (25). Considering the protective effect of albumin in the disease course of MS, higher level of albumin might play more pronounced role. Serum albumin was suggested to be a significant predictor of vitamin D deficiency, thus a lower serum albumin level may suggest possible poorer nutritional management compared to healthy controls (26). Therefore, serum albumin might be more beneficial in this subgroup. Notably, the sample size of females with MS was larger than that of males. Therefore, limited sample size in males with MS might also be a reason that no significant association was identified. In summary, these findings underscore the potential clinical utility of serum albumin as a biomarker for both diagnosis and prognosis prediction in MS, and suggested the potential for targeting albumin to treat MS in clinical trials.

Microalbuminuria is the presence of albumin in the urine above the normal range of less than 30 mg per day, but under the detectable range with the conventional dipstick methodology. The epidemiology of microalbuminuria reveals a close association with systemic endothelial dysfunction and with vascular disease. Patients with MS frequently experience BBB breakdown, microbleeds, reduced cerebral blood flow and diminished neurovascular reactivity, and it is possible that these vascular pathologies are tied to the development of MS (27). Accordingly, it may be expected that the development of microalbuminuria from endothelial dysfunction might be related to MS (27). However, we did not identify association between baseline microalbumin in urine and risk of MS. Consistently, one previous study among 33 patients with MS and 30 controls did not find significant association between urine microalbumin level and risk of MS (27). However, it is noteworthy that the number of incident MS cases was limited in the current study. Consequently, further research is necessary to comprehensively explore the association between microalbuminuria and the risk of MS.

The present study examined the involvement of baseline albumin level in the risk of developing MS. Nevertheless, there are several notable limitations that should be acknowledged. First, the study results were mainly derived from a cohort of Caucasian individuals and may not be generalized to other ethnic populations. Future research endeavors encompassing diverse ancestral cohorts are imperative to validate and extend the observed associations. Second, only albumin in serum and urine were analyzed. Considering that MS is a neurological disorder, exploring the albumin levels in CSF would indeed provide a novel insight.

## Conclusions

The findings from our study suggested elevated levels of pre-morbid serum albumin are associated with a reduced risk of incident MS. These results propose albumin as a potential prognostic biomarker for MS. A deeper understanding of the underlying mechanisms may pave the way for new therapeutic strategies and enable clinicians to intervene effectively, potentially slowing or delaying the progression of the disease.

## Data availability statement

The original contributions presented in the study are included in the article/**Supplementary Material**. Further inquiries can be directed to the corresponding authors.

## Ethics statement

The studies involving humans were approved by West China Hospital Sichuan University. The studies were conducted in accordance with the local legislation and institutional requirements. The participants provided their written informed consent to participate in this study.

## Author contributions

KC: Investigation, Methodology, Software, Visualization, Writing – original draft. CL: Data curation, Formal Analysis, Writing – review & editing. BZ: Supervision, Writing – review & editing. HS: Conceptualization, Funding acquisition, Project administration, Writing – review & editing.
