# Supplementary material for: Albumin and multiple sclerosis: a prospective study from UK Biobank
Source: Front Immunol. 2024 Jun 10;15:1415160. doi: 10.3389/fimmu.2024.1415160 (PMC11194376; doi:10.3389/fimmu.2024.1415160)
Supplement: Supplementary file 1 [file DataSheet_1.docx]

Supplementary Methods

**Details on sociodemographic and lifestyle measures**

Age (data field: 21022): Age at recruitment is a derived variable based on date of birth and date of attending an initial Assessment Center. It refers to the age of the participant on the day they attended an initial Assessment Center, truncated to whole year. Age was adjusted as a continuous variable.

Sex (data field: 31): Sex of participant is acquired from central registry at recruitment, but in some cases updated by the participant. Hence this field may contain a mixture of the sex the NHS had recorded for the participant and self-reported sex. Sex was adjusted as a categorical variable.

BMI (data field: 21001): BMI value is constructed from height and weight measured during the initial Assessment Center visit. Value is not present if either of these readings were omitted. BMI was adjusted as a continuous variable.

Townsend deprivation index (data field: 22189): Neighborhood-level socioeconomic status was measured using the Townsend index of material deprivation. Townsend deprivation index calculated immediately prior to participant joining UK Biobank. Based on the preceding national census output areas. Each participant is assigned a score corresponding to the output area in which their postcode is located. Townsend deprivation index was adjusted as a continuous variable.

Education level (data field: 6138): Education level was measured by touchscreen question "Which of the following qualifications do you have?”. Education level categories were transformed into the following numerical values: “College or University degree” = 20 years; “levels/AS levels or equivalent” = 13 years; “O levels/GCSEs or equivalent” = 10 years; “CSEs or equivalent” = 10 years; “NVQ or HND or HNC or equivalent” = 19 years; “Other professional qualifications, e.g., nursing, teaching” = 15 years; “None of the above” = 7 years; and “Prefer not to answer” = missing. Education level was adjusted as a continuous variable.

Moderate activity (data field: 22038): Metabolic Equivalent Task (MET) minutes per week for moderate activity. Moderate activity was adjusted as a continuous variable.

Vigorous activity (data field: 22039): Metabolic Equivalent Task (MET) minutes per week for vigorous activity. Vigorous activity was adjusted as a continuous variable.

Current tobacco smoking (data field: 1239): Participants were asked about their current tobacco smoking status with touchscreen question “Do you smoke tobacco now?”. Possible answers were reordered to 0 (No), 1 (Only occasionally), and 2 (Yes, on most or all days). Current tobacco smoking status was treated as a categorical variable. Responses of “prefer not to answer” were excluded.

Alcohol frequency (data field: 1558): Individuals were asked by the touchscreen questionnaire: “About how often do you drink alcohol?” and could respond with, “Never”, “Special occasions only”, “One to three times a month”, “Once or twice a week”, “Three to four times a week” or “Daily or almost daily”. If participants felt that this value varied, they were instructed to give an average over the last year. Responses of “prefer not to answer” were excluded. Alcohol frequency was adjusted as a continuous variable.

Vitamin D (data field: 30890): Values measured were measured by CLIA analysis on a DiaSorin Ltd. LIASON XL at based. Vitamin D was adjusted as a continuous variable.

**Supplementary Table 1. ICD-10 and read codes used to identify patients with multiple sclerosis.**

| **ICD-10 code** | **ICD-9 code** | **self-reported disease code** | **Read code V2** | **Read code V3** |
| --- | --- | --- | --- | --- |
| G35 | 340, 3409 | 1261 | 666A., 666B., 8Cc0., 8Cc1., 8Cc2., 8Cc3., 8Cc4., 8CS1., 8Hkv., 9kG.., 9mD.., 9mD0., 9mD1., 9mD2., 9mD3., F20.., F200., F201., F202., F203., F204., F205., F206., F207., F208., F20z. | F20.., F200., F201., F202., F203., F20z., X005c, X005d, X005e, XaKA8, XaKvm, XaKvn, XaKzg, XaOzZ, XaP0r, XaP0X, XaP1B, XaP1V, XaPSa, XaPSb, XaPSc, XaPSY, XaPSZ, XaX9A, XaX9F, XaX9H, XaX9J, XaX9L, XaXsg, XM0eo |
